# Hormonal Dysfunction in Adult Patients Affected with Inherited Metabolic Disorders

**DOI:** 10.34763/jmotherandchild.20202402si.2018.000005

**Published:** 2020-11-10

**Authors:** Karolina M. Stepien

**Affiliations:** 1 The Mark Holland Metabolic Unit, Adult Inherited Metabolic Diseases Department, Salford Royal NHS Foundation Trust, Salford, United Kingdom

**Keywords:** inherited metabolic disorder, endocrine dysfunction, hormones, hypogonadism, thyroid, diabetes mellitus, adrenal failure

## Abstract

Inherited metabolic disorders (IMDs ) are a rare and diverse group of metabolic conditions mainly caused by enzyme deficiencies, and in some of these, hormonal dysfunction is a relatively common complication. It may present in childhood and subsequently hormonal replacement is required throughout their life. Endocrinopathies can be a presenting symptom of an IMD in adulthood, which should be suspected when associated with multiorgan involvement (neurological, musculoskeletal or liver, etc.). A single IMD can affect any gland with hypogonadism, adrenal insufficiency, diabetes mellitus and thyroid dysfunction being the most common. In some cases, however, it is diagnosed later in their adult life as a secondary complication of previous therapies such as chemotherapy used during Haematopoietic Stem Cell Transplantation (HSCT) in childhood.

The mechanisms of endocrine dysfunction in this group of conditions are not well understood. Regardless, patients require ongoing clinical support from the endocrine, metabolic, bone metabolism and fertility specialists throughout their life.

Hormonal profiling should be part of the routine blood test panel to diagnose asymptomatic endocrine disorders with delayed manifestations. It is also worth considering screening for common hormonal dysfunction when patients exhibit atypical non-IMD related symptoms. In some adult-onset cases presenting with multiple endocrinopathies, the diagnosis of an IMD should be suspected.

Given that new therapies are in development (e.g. gene therapies, stem cell therapies, pharmacological chaperone and substrate reduction therapies), clinicians should be aware of their potential long-term effect on the endocrine system.

## Introduction

Inherited metabolic diseases (IMDs) comprise a diverse group of metabolic conditions resulting mainly from enzyme deficiencies. IMDs can be classified into three main groups according to their mechanisms: cellular intoxication, energy deficiency and defects of complex molecules (1).

Although they are individually rare, collectively they have a prevalence of 1 in 784 live births (2). Historically, patients affected with IMDs remained under the care of paediatric metabolic centres; however, most children now survive into adulthood and should be transitioned from paediatric to adult care (3). In addition, attenuated forms of these disorders might not show symptoms during childhood or adolescence, only appearing in adulthood, so an adult service is important. The number of adult patients with an IMD is increasing due to the improved clinical care and advances in the diagnosis. As life expectancy of patients with IMDs has increased, so has the need for specialist services for adults with multidisciplinary care teams, and these should include close links with an endocrine clinic.

Hormonal dysfunction is a relatively common complication of several IMDs. It may present in childhood and subsequently hormonal replacement is required throughout their life. In some cases, however, it is diagnosed later in their life as a *de novo* symptom of the underlying IMD, or as a secondary complication of previous therapies such as chemotherapy used during Haematopoietic Stem Cell Transplantation (HSCT), one of the potential therapies of a specific subgroup of IEM. In some cases, endocrinopathies can indicate a new case of IMD in adulthood, which should be suspected when a condition is associated with multiorgan involvement (neurological, musculoskeletal or liver, etc.) (1). A single IMD can affect any gland, with hypogonadism, adrenal insufficiency, diabetes mellitus and thyroid dysfunction being the most common disorders.

The mechanisms of endocrine abnormalities in this group of conditions are not well understood, however patients require ongoing clinical support from endocrine, metabolic, bone metabolism and fertility specialists throughout their life.

This review describes the mechanisms of endocrine dysfunction in particular IMDs with the aim of raising awareness of this relatively common but underestimated consequence of IMDs (Table 1).

**Table 1:** Endocrine dysfunction in inherited metabolic disorders

Endocrine gland	Endocrine dysfunction	Specific IMD example	Blood tests to screen for the endocrine
Pituitary	Hypopituitarism/Short stature	LSDs: MPS, sialidosis, gangliosidosis, mucolipidosis Others: haemochromatosis, acaeruloplasminaemia, Wilson’s disease, mitochondrial diseases, cystinosis, post-HSCT	TSH, free T4 and free T3, serum cortisol, LH, FSH, male testosterone/estradiol, SHBG, prolactin, IGF1, growth hormone
	Hyperprolactinaemia	GTP cyclohydrolase deficiency, CDGs, GSD Ia	PRL
Thyroid	Hyperthyroidism	Fabry disease (prior to ERT), post-HSCT	TSH, free T4, free T3
	Hypothyroidism	Mitochondrial diseases, cystinosis, Fabry disease, GSD Ib, CGD, hyperoxaluria type I, neutral lipid storage disease	TSH, free T4, free T3, TBG
	Transient thyroiditis	post-HSCT	TSH, free T4, free T3, anti TPO antibodies
Parathyroid	Hypoparathyroidism	LCHAD, MCADD mitochondrial diseases (MELAS) haemochromatosis	Serum calcium, parathyroid hormone, alkaline phosphatase
Ovaries	Hypogonadism	Classical galactosaemia, SLO, CDG, mitochondrial diseases, post-HSCT	LH, FSH, serum estradiol, antimullerian hormone
	PCOS	GSD Ia	LH, FSH, serum estradiol, prolactin
Testicles	Hypogonadism	Haemochromatosis, mitochondrial diseases, cystinosis, X-linked ALD, post-HSCT	Serum testosterone, SHBG, LH, FSH, inhibin B
Adrenal gland	Adrenal insufficiency	X-linked ALD, Lesch-Nyhan syndrome, peroxisomal disorders, Fabry disease, mitochondrial diseases, post-HSCT	Serum cortisol at 9am, short synacthen test, fasting glucose
Pancreas	Diabetes/insulin resistance	Mitochondrial diseases, haemochromatosis, acaeruloplasminaemia, Wilson’s disease, cystinosis, organic acidurias, respiratory chain disorders, GSD I, III and V, Alström syndrome, CDGs	Random glucose, HbA1c, lipid profile, urate

## Hypogonadism and Subfertility

### Haemochromatosis

Primary or secondary hypogonadism is commonly associated with IMDs. Haemochromatosis (HFE) is the most common cause affecting the pancreas, pituitary gland and gonads with an accumulation of iron. As a result, patients present with hypo-or hypergonadotropic hypogonadism. Hypogonadotropic hypogonadism has been found in 5.2% (*n*=38) female patients and 6.4% (*n*=141) male subjects, 89% of whom also have had cirrhosis and 33% diabetes (4). Given that the endocrine abnormality may be the first indicator of an IMD, HFE should always be suspected in male subjects with isolated hypogonadotropic hypogonadism. Pituitary iron overload, as well as the HFE and transferrin genotype, may explain the hypothalamic-pituitary gonadal involvement (5). If diagnosed early, clinical manifestations can be prevented or improved through regular venesection (phlebotomy). The benefits of treatment with androgens has to be carefully balanced in view of their potentially carcinogenic role and increased risk of hepatocarcinoma when associated with cirrhosis (6).

#### X-linked Adrenoleukodystrophy

Hypogonadism may also be observed in X-linked Adrenoleukodystrophy (X-linked ALD), a progressive peroxisomal disorder affecting adrenal glands, testes and myelin stability caused by mutations in the *ABCD1* gene, which encodes for the ALD protein. The anomalies of this peroxisomal transporter induce a ß-oxidation defect of VLCFA (7, 8). X-linked ALD is implicated in more than two-thirds of cases with clinical hypogonadism and anomalies of the gonadotropic axis (9). Severe impairment of spermatogenesis and rapid progression to azoospermia were reported despite normalisation of plasma VLCFA concentrations in a postpubertal patient (10). It was suggested that oxidative stress plays a role, at least in the neurodegeneration features (11).

#### Congenital disorders of glycosylation

Abnormal glycosylation of a variety of proteins involved in hormone metabolism such as transporters, receptors and hormone processing could be the cause of hypogonadism and hypothyroidism in Congenital Disorders of Glycosylation (CDG). The impaired protein glycosylation occurs in the endoplasmic reticulum and Golgi compartments (12).

Elevated tissue fibrosis, observed in CDG patients, could instigate hypogonadism, which is common, especially in males (13, 14). Varying hormonal profiles and degrees of virilisation in CDG-affected females suggest possible unidentified mechanisms affected by impaired *N*-glycosylation (15). In affected women, hypogonadotropic hypogonadism may lead to the absence of secondary sexual development or absence of ovaries (16, 17).

#### Classical galactosaemia

Premature ovarian insufficiency (POI) is a common complication of Classical Galactosaemia (18, 19). Ovarian damage and subfertility is a major complication for females causing a very significant burden of disease. In the majority of adult females with Classical Galactosaemia, POI occurs as a spectrum varying from absent or delayed pubertal development, primary amenorrhoea in adolescents, secondary amenorrhoea or irregular menses to premature menopause (18). There is a lack of insight into the exact timing of the ovarian insult and its pathophysiology (20). The dysregulation of glycosylation, inflammatory response and leptin metabolism as possible cellular event sequences leading to apoptosis have been suggested as possible mechanisms (21).

Follicular stimulating hormone (FSH) levels have been found to be significantly elevated from as early as four months of age (18, 21). The precise timing of the severe decrease in primordial ovarian follicles and the absence of intermediate and Graafian follicles, which suggest a maturation arrest, is not clear (18, 21, 22). There is some evidence that male patients may present with delayed onset of puberty (23) or cryptorchidism (24).

#### Smith-Lemli-Opitz syndrome

Smith-Lemli-Opitz (SLO) syndrome is caused by a deficiency of 7-dehydrocholesterol reductase (DHCR7), the final step in the Kandutsch-Russell cholesterol biosynthetic pathway (25). Inadequate steroid synthesis is the likely explanation of genital morphogenesis and hormonal dysfunction (25). In females, the external genitalia may appear normal or there may be distinct hypoplasia of the labia majora and minora. Premature thelarche and high serum prolactin levels have been previously documented. Menstrual function is often irregular but otherwise normal in most SLO adolescent females and adults, although menarche is often delayed (25). POI was demonstrated in one 25-year-old female (personal observation).

#### Cystinosis

Cystinosis is a lysosomal disease, which induces an intracellular accumulation of cystine due to transportation impairment (1, 26). Hypergonadotropic hypogonadism, which is likely to be related to fibrosis and testicular atrophy, and promotes growth retardation, has been described in 50–75% of males with cystinosis (27, 28, 29). As a result, the onset of puberty is delayed in cystinosis patients with advanced chronic renal disease (27). Azoospermia has been shown to be present in male cystinosis patients treated with cysteamine with spermatogenesis being documented on a testicular biopsy specimen in one renal transplant patient (30). Females with cystinosis have delayed puberty though normal pubertal development is also possible with usually normal fertility in this group (29, 31).

#### Fabry disease

Fabry disease is one of the lysosomal storage disorders (LSDs) caused by lysosomal alpha-galactosidase-A deficiency and is characterised by the systemic accumulation of globotriaosylceramide. A variety of latent endocrine dysfunctions, including life-threatening conditions, have been described in patients with this condition (32). Therefore, an adequate monitoring and hormonal therapy, when required, has to be performed in cases of subclinical endocrine dysfunction to avoid life-threatening events. In one study (33), 89% of women have menstrual disorders (or spontaneous abortions), although gonadotrophic stimulation tests were normal. In males, however, asthenozoospermia has been observed (33).

Screening for possible hormonal dysfunction should be considered before new therapies (e.g. pharmacological chaperone therapy) is commenced. Experimental studies have shown that migalastat increases the risk of infertility in rats, which has not been observed in humans yet (34).

#### Mitochondrial disorders

Mitochondrial diseases are multisystem disorders that feature defective oxidative phosphorylation, and are characterised by enormous clinical, biochemical and genetic heterogeneity. Endocrine dysfunction is a common feature of genetic mitochondrial disease and is associated with decreased intracellular production or extracellular secretion of hormones (35). While diabetes mellitus is the most frequently described endocrine disorder in patients with inherited mitochondrial diseases, other endocrine manifestations include growth hormone deficiency, hypogonadism, adrenal dysfunction, hypoparathyroidism and thyroid disease. Although mitochondrial endocrine dysfunction is a multi-systemic disease, some mitochondrial disorders are characterised by isolated endocrine involvement (35). Hyper- (36, 37) and hypogonadotrophic (38) hypogonadism have been described. Up to 20% of patients with Kearns-Sayre syndrome were affected with hypogonadism (1, 39). In addition, patients with a myopathic component have been found to have anomalies of oestrogen metabolism in the muscle (40).

#### Glycogen storage disorder

Polycystic ovary syndrome (PCOS) associated with hyperinsulinaemia is a common endocrine abnormality in glycogen storage disorder (GSD) type I and III (41) but successful pregnancies have been described (42). The alterations in glucose metabolism play a probable role in the ovarian pathophysiology. Delayed puberty-related growth impairment and short stature have also been documented.

#### Wilson’s disease

Hypogonadotropic hypogonadism secondary to chronic liver disease is the most common cause of gonadal dysfunction in patients with Wilson’s disease. In addition, excessive sex hormone binding globulin production, elevated prolactin levels and direct suppression of Leydig cell function also contribute to gonadal dysfunction in these patients (47).

#### Hypopituitarism and growth

Growth retardation is multifactorial in patients with IMDs and can be attributed to hypopituitarism in mitochondrial cytopathies and iron-overload diseases. Other disease-specific complications, e.g. renal failure or liver dysfunction, poor nutrition or psychosocial issues, may indirectly contribute to significant growth retardation (89). Up to 60% of patients with mitochondrial cytopathies, cystinosis (30) and classical galactosaemia (43) have been found to have short stature (1). It is a common feature of the LSDs including Mucopolysaccharidosis (MPS) (44) or Niemann Pick B (45). Delayed skeletal maturation, psychosocial morbidity and low Insulin-like Growth factor 1(IGF1) concentration may be contributing factors (26). Growth hormone replacement has been trialled in MPS patients with variable success rate (personal observation).

Schindler’s disease, caused by a mutation in the *NAGA* gene causing alpha-N-acetylgalactosaminidase enzyme deficiency, affects breaking down of glycoproteins and glycolipids. Short stature, despite normal hormonal profile, and learning difficulties are some of the features. Gangliosidosis GM1, caused by deficient beta-galactosidase activity, is characterised clinically by a wide range of variable neurovisceral, ophthalmological and dysmorphic features. Short stature is a common dysmorphic feature; although hormonal dysfunction has not been proven so far, possible alterations in the growth hormone/IGF1 pathways have been considered (46).

#### HSCT and hypogonadism

HSCT has been established as an effective therapy for selected IMDs, including some LSDs. This group encompasses over 70 diseases, which comprise genetic defects in specific lysosomal proteins. Given their complexity, all LSDs require multi-disciplinary management to optimise treatment response and prevent premature mortality (48). The function of lysosomes is now thought to extend beyond their involvement in degradation and recycling of extracellular and intracellular material. They play a crucial role in plasma membrane repair, lipid and metabolite exchange between organelles and have recently been found to regulate energy metabolism via calcium signalling (48, 49).

The HSCT has been shown to be particularly successful in the early treatment of MPS Type IH (Hurler’s syndrome) (48). It has been a standard or optional method of treatment for several other IMDs including MPS II, VI, Tay-Sachs disease or X-linked ALD. HSCT rather prevents disease progression than reversing already established disease manifestations. This treatment requires full intensity myeloablative conditioning, e.g. with fludarabine and pharmacokinetic-guided busulfan dosing being the current recommendation for LSDs (50). However, in the recent past, high doses of cyclophosphamide and busulfan were used, which in combination with total body irradiation caused long-term endocrine complications. Most adult female patients who underwent HSCT in childhood present with POI, whereas most males have compensated hypergonadotropic hypogonadism. It is believed that with the new busulfan pharmacokinetics monitoring, the long-term endocrine dysfunction may become less common.

Hypothyroidism or transient thyroiditis has also been observed post HSCT. A patient affected with juvenile Tay-Sachs who underwent HSCT at the age of 15 years and an MPS IH patient required regular monitoring in a joint metabolic and endocrine clinic and received hormonal replacement (personal observations). Low serum corticol with normal short synacthen tests have been observed in adult MPS IH patients who underwent HSCT in childhood.

Although the survival of patients affected with IMD has improved, hypogonadism remains one of the most frequent complications in adolescence and adulthood. Early diagnosis and hormonal replacement enables pubertal progress and reduction in growth retardation, bone loss in adulthood and related mental health issues. The onus is on adult metabolic teams to screen for hypergonadotrophic (primary) or hypogonadotrophic (secondary) hypogonadism and osteoporosis.

In general, we recommend annual screening for endocrine dysfunction in this group of patients. Early diagnosis and replacement of hormonal deficiencies are critical for general well-being and bone health in adulthood.

## Thyroid

### Mitochondrial disease

The underlying mechanisms by which thyroid abnormalities occur are complex and incompletely understood. It is suspected that a lack of adenosine triphosphate production and/or increased oxidative stress in endocrine cells with impaired mitochondria may lead to failure of hormonal synthesis and/or secretion (35). A few associations with specific nuclear encoded genetic defects have been noted, particularly in *TANGO2* and *PTRH2* mutations, but insights about the presence of a causal mechanism between mitochondrial disease and thyroid dysfunction are lacking. The overall prevalence of hypothyroidism, the most common thyroid abnormality, has been estimated as 6.3% (51) and increases with age. Goitre has also been reported. Energy defects could impair thyroperoxidase, lowering the production of thyroid hormones, which themselves are involved in mitochondrial metabolism, therefore worsening the mitochondrial dysfunction (1).

### GSDs

Several IMDs are associated with dysthyroidism in adulthood and mainly involve energy metabolism defects and the degradation or synthesis of complex molecules. Given that GSD Ib is associated with neutropenia that increases the risk of autoimmune infections, there is an increased prevalence of autoimmune hypothyroidism with raised thyroid-stimulating hormone (TSH), thyroglobulin levels and antithyroperoxidase antibodies (52). Another possible pathomechanism of abnormal thyroid function tests in patients with GSD is their chronic liver disease. There has been a negative correlation between free T3 levels and direct bilirubin, suggesting an association between the disease severity and the thyroid function test (53). Euthyroid sick syndrome or subclinical hypothyroidism was documented in GSD patients with chronic liver diseases (53).

### Neutral lipid storage disease

This rare non-lysosomal lipid storage disorder, caused by defects in two triglyceride-associated proteins, results in impaired degradation of triglycerides and causes their accumulation in cells with two different phenotypes (54). Apart from myopathy, cardiomyopathy, ichthyosis, hepatomegaly or central nervous system involvement, thyroid nodular dystrophy related to intracellular accumulation of triglycerides have been reported (55). Abnormal lipid metabolism may lead to other endocrine abnormalities such as low leptin and adiponectin levels, a moderate increase in insulin levels at fasting state and even greater increase after oral glucose tolerance test (56).

### CDG

Thyroid function tests are frequently abnormal in children with CDG (17, 57), in whom congenital hypothyroidism has been suspected. In adults with CDG, clinical hypothyroidism is rare (1). As part of the follow-up protocol, thyroid hormones including TSH, free T3 and free T4 should be measured. Low thyroxine-binding globulin (TBG) and transient elevations in TSH were previously documented (17, 57).

### Fabry disease

Subclinical dysthyroidism is frequent and resolves with enzyme replacement therapy (ERT). Subclinical, non-autoimmune hypothyroidism was observed in 30% of cases in an untreated series (32). In some cases, however, undiagnosed hyperthyroidism may aggravate adverse reactions to ERT (32). It highlights the importance of requesting hormonal profile in all patients before ERT is considered (personal observations).

### Cystinosis

Progressive cystine accumulation and crystal formation in thyroid follicular cells causes fibrosis and atrophy leading to primary hypothyroidism in 50–75% of patients with cystinosis (1, 28), manifesting in the majority of patients from the second decade of life (29). Impaired thyroglobulin synthesis and iodo-thyroglobulin processing might be responsible for subclinical hypothyroidism with TSH elevation and normal T3 and T4 plasma concentrations (29). Importantly, HSCT has been shown to correct thyroid disease in mice (58), which was not documented in human beings.

## Adrenal Insufficiency

Adrenal insufficiency has been documented in several different IMDs. If undiagnosed and not treated, it can be life threatening. Therefore, screening by requesting a morning serum cortisol and if abnormal, verifying the abnormality with a short synacthen test is a part of a routine protocol in our joint endocrine/metabolic clinic.

### X-linked ALD

The mechanisms of adrenal dysfunction vary. This common finding in X-linked ALD is caused by defective catabolism resulting in VLCFA accumulation, which interferes with steroid hormone synthesis. In adulthood, adrenal insufficiency, which generally appears after the age of 3–4 years, is present in 70% of patients. It can be the first and only manifestation of the disease for decades. Its association with neuropathy suggests the diagnosis. At the age of 20, it is usually associated with hypergonadotropic hypogonadism (1).

### Peroxisomal disorders

Importantly, the peptide hormones β-lipotropin (β-LPH) and β-endorphin were found to be localised to peroxisomes in various human tissues (59). This suggests a functional link between peptide hormone metabolism and peroxisomes. In addition, it has been postulated that peroxisomes are involved in steroidogenesis because patients affected with peroxisomal disorders have endocrine manifestations that affect steroid hormones (59).

### LSDs

Adrenal dysfunction is a common complication of several LSDs and results from storage material accumulation in adrenal glands, e.g. Fabry disease (60), cystinosis (30) or Niemann Pick B; a non-neurologic, visceral form with hepatosplenomegaly, pulmonary disease and survival into adolescence and/or adulthood (45).

### Mitochondrial disorders

Mitochondrial disease-related adrenal insufficiency is rare in childhood; however, this may be the first symptom (60) and is associated with poor prognosis (1, 61). In adulthood, subclinical adrenocortical insufficiency is sometimes found in multisystemic forms as shown in cases affected with POLG mutation (62). Various types of aldosterone secretion disturbances, mainly secondary hyperaldosteronism possibly associated with tubulopathies, have also been reported (63).

### Lesch-Nyhan syndrome

Lesch-Nyhan syndrome, a rare X-linked recessive disorder of purine synthesis, is characterised by the absence of hypoxanthine guanine phosphoribosyl transferase (HPRT). The adrenal medulla may be directly affected by the absence of HPRT enzyme. Polyendocrinopathy is typical of this condition and has been previously documented (64). The deficiency of the normally high activities of HPRT in the testes appears to inhibit their ability to respond to gonadotrophin (64). Boys often present with growth delay, testicular atrophy and a partial failure of the 11 beta-hydroxylation of steroids (64). Persistently low cortisol with satisfactory short synacthen test has been previously reported although mechanisms were unclear (personal observation).

### SLO

Adrenal insufficiency is a common endocrine complication of SLO syndrome (65, 66). It is recommended that all patients affected with this condition have morning serum cortisol and pituitary gland hormones and electrolytes checked prior to any surgical intervention and during the acute illness to ensure early intervention to correct these biochemical consequences, to reduce mortality and to improve long-term outcome (66).

## Insulin Dysregulation

The mechanisms of diabetes development in IMDs involve either defects of insulin secretion or insulin resistance, which might be promoted by liver or muscle involvement and oestrogen deficiency (67). Other mechanisms involve insulin deficiency resulting from impaired pancreatic ß cell function (1). Iron deposition in pancreas in HFE or aceruloplasminaemia (1) and ketoacidosis-induced pancreatitis in organic acidaemias (1, 68, 69), may present with diabetes.

### HFE and aceruloplasminaemia

Hereditary HFE caused by a mutation in *HFE 1* gene is one of the most frequent metabolic diseases and the penetrance is variable and modulated by environmental factors such as body weight and alcohol.

Non-transferrin-bound iron plays an important role in cellular iron damage through an increase in oxidative stress. First, liver iron overload promotes the occurrence of insulin-resistant diabetes (70). The pancreas ß cells may then be progressively destroyed, leading to C-peptide negative diabetes and requiring insulin therapy besides iron depletion.

Aceruloplasminemia is characterised by the accumulation of iron in the liver, islets of Langerhans and the brain, related to decreased cellular iron egress, in contrast with most other types of iron overload (71). The treatment with fresh frozen plasma infusion and iron chelation is efficient in reducing iron overload and preventing both neurological involvement and diabetes in this condition (personal observation).

### GSDs

Disorders of energy metabolism such as GSDs comprise the other mechanisms of diabetes development in IMDs. GSDs are associated with the highest number of endocrine anomalies. Glycogen deposits and secondary glycosylation disturbances in GSDs (in which only the pancreas and ovaries are affected) are the most probable mechanisms involved in these disorders (1).

GSD is the result of defects in the processing of glycogen synthesis or breakdown within muscles, liver and other cell types. Type I GSD is initially associated with severe fasting hypoglycaemia.

Postprandial hyperglycaemia can occur in adulthood (1) and is likely to be associated with recurrent pancreatitis and hypertriglyceridaemia, which lead to insulin secretion impairment or an adaptive mechanism of the glucose receptor, GLUT2, for reducing the secretion of insulin. Diabetes is also promoted by insulin resistance related to liver and/or muscle dysfunction (72, 73).

Apart from GSD type I and III, diabetes mellitus has become a common feature of GSD V (McArdle syndrome) with 30% of adult patients affected with this condition (personal observation).

### Cystinosis

Endocrine and exocrine pancreatic insufficiency is a long-term complication of cystinosis, an organelle disorder, usually after renal allograft transplantation (29). Up to 50% of infantile cystinosis patients by the second decade of life develop slow progressive loss of insulin secretion and C-peptide production leading to glucose intolerance and diabetes mellitus (1, 29, 74; 87). These complications are only partially prevented or improved with cysteamine treatment (1).

### Mitochondrial diseases

Mitochondrial diabetes accounts for 0.06–2.8% of cases of type 2 diabetes. The diagnosis should be considered in young, lean patients with non-autoimmune diabetes and neurosensory or muscular involvement (1). A higher level of heteroplasmy has been found in mitochondrial diabetes in the endocrine pancreatic ß cells than in the exocrine cells and blood leucocytes (75).

There are different forms of mitochondrial diabetes (76, 88) and may be associated with multiple endocrine dysfunctions. In mitochondrial diseases impaired pancreatic ß cell function is a result of ATP deficiency (77). The production of ATP through the glycolysis pathway, too low to ensure normal cell function, leads to cell death, as confirmed histologically by a reduction in the number of ß cells (1, 78). It also prevents closure of the potassium channels, inducing a defect in insulin secretion (79). Insulin sensitivity in skeletal muscle is also decreased (79). As a result, insulin requirement may increase with occasional ketoacidosis. Proteinuria and renal insufficiency occur more frequently than usual in diabetes (1).

### CDG

Endocrine abnormalities including hyperprolactinemia, growth hormone release with hyperglycaemia, insulin resistance, and hyperinsulinemic hypoglycaemia have been described in CDG (16, 17). A recent review by Moravej et al. confirmed that hypoglycaemia is associated with hyperinsulinism in 43% of phosphomannomutase 2 deficiency (PMM2-CDG) patients (80). Most patients with phosphomannose isomerase deficiency (MPI-CDG) develop hypoglycaemia, but due to the often-severe gastrointestinal symptoms, moderate episodes of hypoglycaemia may be compensated for or masked by frequent enteral and parenteral nutrition (80, 81). Most patients with phosphoglucomutase 1 deficiency (PGM1-CDG) develop hypoglycaemia, which seems to be because of multifactorial: hyperinsulinism, inappropriate counter-regulatory hormonal response (low cortisol), impaired glucose release from glycogen during fasting and malnutrition (82). Most cases are managed conservatively, but in some others, diazoxide was used (80).

### Wilson’s disease

Diabetes has occasionally been described in patients with Wilson’s disease. Excessive fat deposition in liver and nuclear glycogen deposition contributing to hepatic insulin resistance has been postulated in these individuals (47).

### Alström disease

This rare disorder resulting from a defect in ALMS1 protein localised to the centrosomes and basal bodies of ciliated cells is characterised by short stature, renal failure, dilated myocardiomyopathy, blindness and deafness (1, 83). About 92% of patient with this syndrome develop insulin resistance before the age of 16, obesity and primary hypogonadism (in males) and PCOS and hirsutism in females (1).

### Hypoparathyroidism

Some mitochondrial disorders such as mitochondrial myopathy, encephalopathy, lactic acidosis and stroke-like episodes (MELAS) due to point mutations in mitochondrial tRNA; long-chain 3-hydroxyacyl-CoA dehydrogenase (LCHAD) or combined mitochondrial trifunctional protein (MTP) deficiency due to mutations in MTP (84); medium-chain acyl-CoA dehydrogenase deficiency (MCADD) due to mutations in the *ACADM* gene (85, 86). The mechanism by which these mitochondrial defects affect parathyroid gland development or function is unknown (86).

No obvious endocrine consequences have been described so far in aminoacidopathies or urea cycle disorders (1).

### Conclusions

IMDs are commonly associated with endocrinopathies, which remain undiagnosed in many cases. Hormonal profiling should be part of the routine blood test panel to diagnose asymptomatic endocrine disorders with delayed manifestations. It is worth considering screening for common hormonal dysfunction when patients are symptomatic. In some adult-onset cases presenting with multiple endocrinopathies, the diagnosis of an IMD should be suspected.

Given that new therapies are in development, e.g. gene therapies, stem cell therapies, pharmacological chaperone and substrate reduction therapies, clinicians should be aware of their potential effect on the endocrine system.

## Abbreviations

CDG: congenital disorders of glycosylation
ERT: enzyme replacement therapy
HFE: haemochromatosis
HSCT: haematopoietic stem cell transplantation
IMD: inherited metabolic disorders
FSH: follicular stimulating hormone
GSD: glycogen storage disorders
LCHAD: long –chain 3-hydroxyacyl- acyl-CoA dehydrogenase deficiency
LH: luteinising hormone
LSD: lysosomal storage disorders
MPS: mucopolysaccharidosis
PCOS: polycystic ovaries syndrome
POI: premature ovarian insufficiency
SHBG: sex hormone binding globulin
SLO: Smith-Lemli-Opitz syndrome
TBG: thyroxine-binding globulin
TSH: thyroid-stimulating hormone
VLCFA: very long chain fatty acid
X-linked ALD: X-linked Adrenoleukodystrophy.
